# Modern Medicine: Its Theory and Practice

**Published:** 1910-06

**Authors:** 


### Books and Magazines.

MODERN MEDICINE: ITS THEORY AND PRACTICE. In original contributions by American and foreign authors. Edited by William Osler, M. D., Regius Professor of Medicine in Oxford University, England; formerly Professor of Medicine in Johns Hopkins University, Baltimore; in the University of Pennsylvania, Philadelphia, and in McGill University, Montreal. Assisted by Thomas McCrea, M. D., Associate Professor of Medicine and Clinical Therapeutics in Johns Hopkins University, Baltimore. In seven octavo volumes of about 900 pages each, illustrated. Volume VII, Diseases of the Nervous System, Mental Diseases, General Index. Just ready. Price per volume, cloth, $6.00, net; leather, $7.00, net; half Morocco, $7.50, net. Lea & Febiger, Publishers, Philadelphia and New York.

The publication of the seventh volume completes the greatest work on practical medicine ever offered to the profession. Dr. Osler's pre-eminent position has enabled him to secure articles from the leaders in every department. Their co-operation under a skillfully devised plan has resulted in the creation of a complete library of present-day medicine within the convenient compass of about six thousand pages, adequately illustrated. Each volume is indexed, and the seventh contains a general index to the whole, so that the latest authoritative information on any point is immediately at command. For a work of such paramount value and usefulness a phenomenal demand was assured, and it was, therefore, possible to fix a price low enough to meet the convenience of every practitioner who aims to qualify in full measure for his responsibilities.

